# Sensory, Physico-Chemical and Water Sorption Properties of Corn Extrudates Enriched with Spirulina

**DOI:** 10.1007/s11130-017-0628-z

**Published:** 2017-09-02

**Authors:** Małgorzata Tańska, Iwona Konopka, Millena Ruszkowska

**Affiliations:** 1 Faculty of Food Sciences, University of Warmia and Mazury, Pl. Cieszyński 1, 10-726 Olsztyn, Poland; 2 Department of Commodity Science and Quality, Maritime Academy, Ul. Morska 83, 81-225 Gdynia, Poland

**Keywords:** Spirulina, Extrusion-cooking, Quality of extrudates, Water sorption isotherm

## Abstract

This study compares the quality of extrudates made from corn grits with the addition of up to 8% of spirulina powder. The sensory properties (shape, color, aroma, taste and crispness), chemicals (content of water, protein, fat, ash, fiber, carbohydrates, carotenoids, chlorophyll and phycocyanin) and physical properties (color, water absorption index, expansion indices, texture and water sorption properties) were determined. It has been found that spirulina-enriched extrudates had slightly lower sensory scores, but the addition of spirulina improved their nutritional value. The contents of protein, ash, fiber and β-carotene increased in extrudates with 8% of spirulina by 34, 36, 140 and 1,260%, respectively. The increasing addition of spirulina caused a decrease in extrudates lightness, an increase in their greenness and yellowness accompanied by a decrease of expansion indices and an increase of softness. Only small differences were found in water sorption properties, suggesting a similar behavior of spirulina-enriched extrudates during storage.

## Introduction

Spirulina biomass is popular as a dietary and feed supplement in the aquaculture, aquarium and poultry industries [1]. It contains ca. 70% of protein (with all essential amino acids), ca. 15–20% of carbohydrates (composed of glucose and glycogen), and ca. 7–11% lipids (ca. 70–80% as free fatty acids) [2]. Approx. 20% of protein constitutes phycocyanin, which is an intensively blue biliprotein [3]. This colorant is highly stable in the pH range of 5–8 and is known as a natural food colorant [4]. Spirulina is also extremely abundant in carotenoids (β-carotene), B vitamins (B_1_, B_2_, B_3_), iron, calcium, phenolic acids and tocopherols [1–3]. Based on its unique composition, spirulina has been called a “superfood” [3]. It may be consumed as a whole food, but it is better tolerated in the form of various drinks and tablets [1, 3]. It may have immunemodulatory, antiinflammatory, anticancer, antiviral, antiallergic, and cholesterol-lowering effects, without any significant side-effects [5]. It also protects against radiation and malnutrition, heavy metal intoxication, obesity, diabetes, depression and other body dysfunctions [2]. Its pro-health properties are highly related to phycocyanin activity [5].

Spirulina may be utilized for the production of pasta and various breads and crisps. Rodríguez De Marco et al. [6] determined the effects of its addition on the quality of bread wheat pasta and showed that only pasta with at least 20 g of spirulina/100 g of flour was slightly modified in quality. The incorporation of spirulina resulted in an increase in protein content; accompanied by slightly reduced protein digestibility. Pasta with spirulina exhibited also a higher phenolics content and antioxidant activity. Selmo and Salas-Mellado [7] reported that an increase in this alga from 1 to 4% in formulation of rice flour bread did not affect the preference of the consumer judges. Spirulina may be also used as a valuable component of extrusion-cooking products. Pure (basic) corn puffs are of relatively low nutritious value. For this reason, they often are enriched by various nutritionally-valuable additives [8]. Spirulina was also utilized in this approach. For example Joshi et al. [9] after optimization of extrusion process parameters and blend composition incorporated up to 7.5% spirulina into puffs, which were of increased protein, carotenoids and zinc contents. Using supercritical fluid extrusion, Bashir et al. [10] produced spirulina-fortified (up to 6%) rice-soy crisps and recorded their high acceptability by sensory judges. It seems that spirulina biomass may be successfully used for the production of extrudates. Even a small addition of this alga substantially increases the protein and many other physiologically active ingredients [10]. The allowable amount is generally dependent on sensory acceptance by consumers and usually do not exceed 8%. The quality and acceptability of such products may be affected by the production technology (single- or twin-screw extrusion, processing parameters, etc.) and may be changed during storage, since extrudates are highly hygroscopic.

The main aim of this study was to determine the effect of spirulina powder addition on corn-enriched extrudates quality and water sorption characteristics during storage under various relative humidity levels. Additionally, the retention of main spirulina pigments (phycocyanin, carotenoids, and chlorophylls) under the extrusion process was studied.

## Materials and Methods

The basic materials were corn grits with particle size in range of 0.36–0.65 mm (Sante, Sobolew, Poland) and spirulina powder with particle size <0.15 mm (Bio Organic Foods, Białystok, Poland) bought in local markets. The extrusion-cooking process was conducted using an S 45A-12-10 U single-screw extruder (Metalchem, Gliwice, Poland) (screw speed 125 rpm, screw length/diameter ratio 12:1, compression ratio 3:1, die diameter 4.5 mm, feed rate 25 kg/h). The temperature distribution in the extrusion-cooker barrel was 105 °C/130 °C/110 °C. Six types of mixtures (each of 1 kg) were prepared: control (corn grits), corn grits with 2, 4, 6 and 8% of spirulina powder and corn grits with 8% of spirulina powder +2% of baking powder (by manual mixing to uniform mass). The moisture content of all prepared mixtures before extrusion was adjusted to 14%. Experiment was carried out in two runs for each variant. Fresh extrudates were cooled (4 h at 19±2°C and relative humidity of 54±2%), packed in plastic bags and stored in cool and dry place until analyzes.

The composition of corn grits, spirulina powder and the extrudates was determined according to AOAC [11] standards: moisture (Method 930.15), protein (Method 990.03), fiber (Method 962.09), fat (Method 920.39, using hexane), ash (Method 942.05), while carbohydrates were determined as the rest of dry mass to 100%. The pigment content was determined spectrophotometrically: chlorophyll a and β-carotene were extracted with 80% acetone [12] and phycocyanin with deionised water [13].

A consumer analysis of sensory quality of the extrudates was carried out in a sensory evaluation laboratory using a 5-point scale, where 1 point represented the lowest level of acceptance and 5 points was the highest [14]. The panel consisted of 20 persons who tested five sensory attributes such as shape, color, aroma, taste and crispness.

**Fig. 1:**
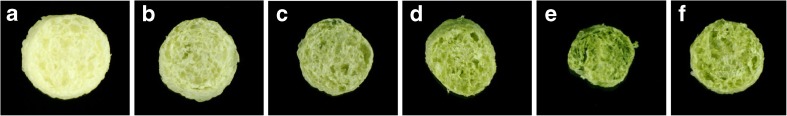
Cross-section of extrudates: control corn (**a**) and with 2% (**b**), 4% (**c**), 6% (**d**), 8% (**e**) of spirulina, and with 8% of spirulina and 2% of baking powder (**f**)

The color of extrudate cross-sections (Fig. 1) was measured with a Digital Image Analysis set and expressed in CIEL*a*b* color system [15]. Images were acquired with a Nikon DXM-1200 (Nikon Inc., Melville, USA) charge-coupled device (CCD) color camera. The color parameters were designated using LUCIA G v. 4.8 software (Laboratory Imaging, Prague, Czech Republic). The light source was a Kaiser RB 5004 HF – High Frequency Daylight Copy Light set with 4 × 36 W fluorescent tubes of 5400 K) (Kaiser Fototechnik GmbH & Co. KG, Germany). The expansion of the extrudates was characterized based on the expansion ratio (the ratio of the diameter of the extrudate to the die diameter) and the longitudinal and radial expansions (the linear length and diameter of extrudates measured with a caliper). The specific density of the extrudates was determined by the weight-to-volume ratio of individual extrudates [16]. The mechanical properties were determined by a universal testing machine (model 4301, Instron Corp., Canton, MA, USA) using a uniaxial compression test. Extrudates were compressed with a constant rate of 50 mm/min and the maximal force (hardness) was recorded to the assumed 50% deformation. The breaking stress was evaluated as the force divided by the cross-section area of the extrudates [17].

The storage durability was determined by evaluation of sorption isotherms and isotherm parameters [18]. The extrudates were put into a hygrostat with a water activity (a_w_) in the range of 0.07–0.98 and stored for a period of 45 days at 25 °C. The mathematical descriptions of sorption isotherms were made based on the BET (Brunauer, Emmett and Teller) equation in the range of a_w_ = 0.07–0.33. The extrudates monomolecular layer capacity, the sorption-specific surface, energy constant, total capacity and the size of capillaries were determined [18]. The results were analyzed using Jandel-Table Curve 2D software v. 5.01. The fit of the empirical data to the BET equation was characterized by the determination coefficient (R^2^) and the standard error estimation (FitStdErr) and Fstat statistics.

The results of all analyses were analyzed using Statistica 12.0 PL software (StatSoft, Kraków, Poland). The differences between the means were determined using analysis of variance (ANOVA) at a significance level *p* = 0.05 with Tukey’s test.

## Results and Discussion

**Fig. 2:**
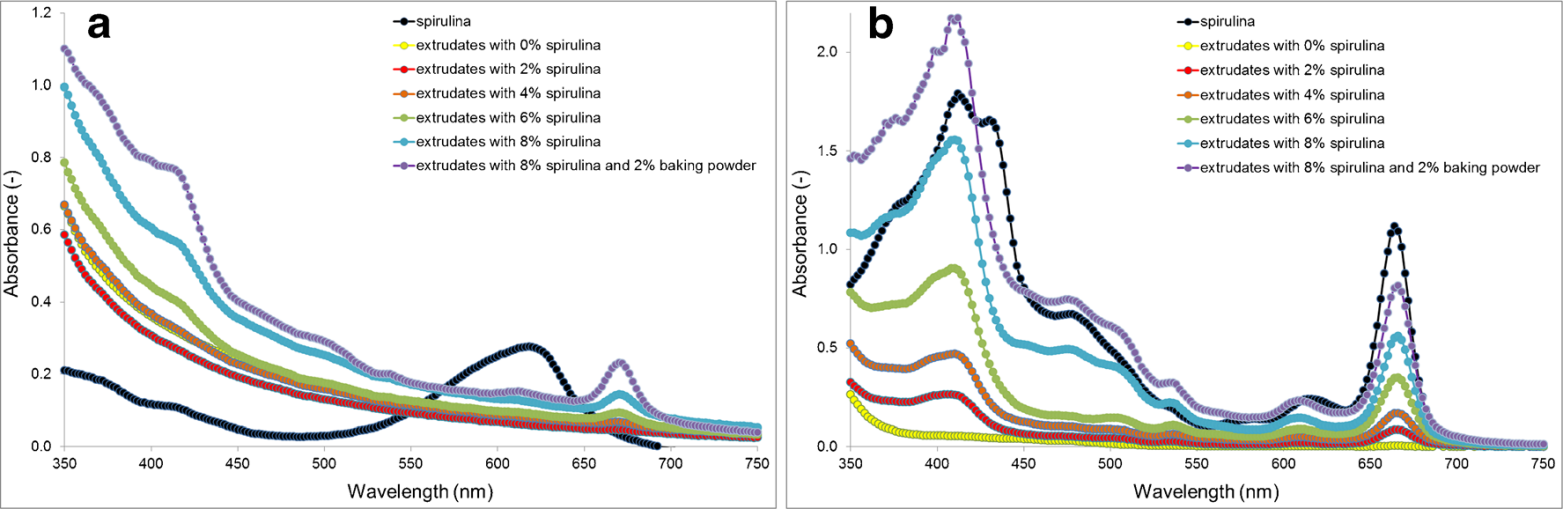
Absorption spectra of water (**a**) and acetone (**b**) extracts (concentration: 0.2% of spirulina powder and 5% of extrudates, *w*/*v*)

It was found that the extrudates were similar in initial moisture content (variation in range of 6.4–6.6%). Since spirulina contained 60.7% of protein, the extrudate protein gradually increased from 11% in the control sample to 15.7% in the extrudate with 8% of spirulina (Table 1). The average increase in protein content was approx. 0.6% per each percentage of added spirulina. Similarly, an increase in fat and ash (both approx. 0.1% per percentage of spirulina) and fiber (approx. 0.8% per percentage of spirulina) was found. In summary, an addition of 8% spirulina resulted in an increase in protein (+4%), ash (+0.4%), fat (+0.4%) and fiber (+0.7%). These increases were accompanied by a decrease in total carbohydrates from 79.4% to a final content of 74.3%. Enriched extrudates were also abundant in carotenoids and chlorophylls (up to 8.8 and 27.5 mg in 100 g, respectively), but these amounts were only 52 and 37% in relation to found in mixture before extrusion. In a study by Joshi et al. [9], the changes in carbohydrates, protein and fat after a 7.5% addition of spirulina were similar, while the carotenoid increase was approximately 10-fold in relation to the control corn extrudate (in our study was close to 18-fold). However, the phycocyanin content of extrudates was extremely low (only traces). This was due to the effect of extrusion-cooking, since this compound is sensitive to temperature. The critical temperature for the stability of phycocyanin was assayed as 47 °C [19]. To confirm the impact of extrusion on pigments we analyzed water (soluble blue phycocyanin) and acetone extracts (β-carotene and chlorophyll) of native spirulina and extrudates. An analysis of water extracts (Fig. 2a) showed that extrusion caused the complete degradation of phycocyanin (ca. 615 nm), accompanied by the appearance of water-soluble chlorophyll-like compounds [20, 21]. This type of chlorophyll derivative may be formed by a loss of magnesium and its replacement by sodium/cooper and the creation of salts [19]. Extrusion caused a significant reduction of carotenoids and chlorophylls (Fig. 2b). Interestingly, in acetone extract the absorption band in the region characteristic for phycocyanin was also found. The strong protective effect of baking powder on spirulina pigments retention in extrudates was found.

**Table 1:** Chemical composition of raw materials and extrudates

Chemical component	Corn grits	Spirulina powder	Extrudates					
			Control	2% S	4% S	6% S	8% S	8% S + 2% BP
Moisture (%)	11.4	5.4	6.5	6.5	6.5	6.4	6.4	6.6
Protein (% d.b.)	11.1	60.7	11.7	12.7	13.7	14.7	15.7	15.7
Carbohydrates (% d.b.)	75.2	15.2	79.4	78.1	76.9	75.7	74.3	74.1
Fibre (% d.b.)	0.5	7.9	0.5	0.7	0.8	1.0	1.2	1.2
Fat (% d.b.)	1.0	7.2	1.1	1.2	1.3	1.4	1.5	1.5
Ash (% d.b.)	1.3	7.9	1.4	1.5	1.6	1.8	1.9	2.0
Pigments (mg/100 g d.b.)								
Phycocyanin	0.0	2446.0	0.0	0.0	0.0	0.0	tr.	tr.
Chlorophyll a	0.0	703.5	0.0	3.2	6.2	12.6	20.8	27.5
β-carotene	0.6	156.5	0.5	0.8	1.1	1.5	6.8	8.8

*d.b.* dry basis, *S* spirulina powder, *BP* baking powder, *tr.* traces; *n* = 3

The sensory panel classified all produced extrudates as accepted for consumption with an overall score from 3.9 to 4.9 points on a 5-point scale (Table 2). The highest score was noted for control extrudates and its value decreased along with a gradual increase in spirulina powder addition. The most diminished features were color and crispness, with scores of 3.7 and 3.8 for an 8% spirulina addition. Although the color of enriched extrudates was generally accepted by consumers, the increasing addition of spirulina powder caused a linear decrease of their lightness (from 86.9 to 61.4%), accompanied by an increase in their greenness (from −3.8 to −5.7) and yellowness (from 21.4 to 34.9). The total color differences between extrudates in the CIELab space (in relation to control sample) were 10.3, 16.8, 22.0 and 26.2, for 2, 4, 6 and 8% of spirulina, respectively (Table 3). The control extrudate was mostly darkened and discolored in comparison to raw material, while the lightness of spirulina-enriched extrudates tended to retain these values and to increase the saturation with green and yellow. The high impact of a 2% addition of baking powder on extrudate color is also noteworthy. The L* and a* attributes of this sample were similar to the variant using an addition of 2% of spirulina, while the value of the b* attribute was the highest among the tested variants. These significant (visible) differences can be also seen in a photo of the obtained extrudates (Fig. 1).

**Table 2:** Sensory characteristics of extrudates (number of points)

Sensory feature*	Extrudates											
	Control		2% S		4% S		6% S		8% S		8% S + 2% BP	
	x	SD	x	SD	x	SD	x	SD	x	SD	x	SD
Shape	4.9	0.2	4.8	0.3	4.4	0.1	4.2	0.2	4.3	0.2	3.8	0.3
Color	4.9	0.1	4.7	0.2	4.6	0.2	4.5	0.3	3.7	0.4	3.7	0.4
Aroma	4.9	0.0	4.5	0.3	4.3	0.5	4.2	0.4	4.0	0.3	3.9	0.3
Taste	5.0	0.2	4.7	0.3	4.5	0.5	4.2	0.4	4.0	0.4	3.7	0.3
Crispness	4.8	0.1	4.7	0.3	4.3	0.4	4.0	0.5	3.8	0.2	4.2	0.2
Overall score	4.9	0.1	4.7	0.3	4.4	0.3	4.2	0.4	4.0	0.3	3.9	0.3

*S* spirulina powder, *BP* baking powder; *n* = 20

*Each feature was designated in 5-point scale, where 1 point – the least accepted, and 5 points – the most accepted

**Table 3:** Color of raw material mixtures and extrudates

Color attribute	Sample											
	Control		2% S		4% S		6% S		8% S		8% S + 2% BP	
	x	SD	x	SD	x	SD	x	SD	x	SD	x	SD
Raw material mixtures												
L* (%)	93.2	1.0	78.7	0.8	71.5	1.2	65.5	0.6	61.1	1.4	62.4	0.7
a* (−)	−9.6	0.4	−5.0	0.5	−3.8	0.3	−3.2	0.4	−2.4	0.2	−2.4	0.2
b* (−)	58.2	1.1	37.4	1.3	30.0	0.6	24.4	0.2	21.8	0.8	20.1	1.2
ΔE (−)	–	25.8	36.1	44.2	49.1	49.5						
Extrudates												
L* (%)	86.9	1.9	76.7	1.5	70.4	0.5	65.6	1.1	61.4	0.6	74.0	0.8
a* (−)	−3.8	0.3	−4.5	0.3	−4.9	0.1	−5.5	0.3	−5.7	0.1	−4.1	0.3
b* (−)	21.4	0.7	22.7	0.2	24.2	1.4	26.7	1.4	27.0	0.5	34.9	1.0
ΔE (−)			10.3		16.8		22.0		26.2		18.7	

*S* spirulina powder, *BP* baking powder, *ΔE* total color difference calculated from CIEL*a*b* space from the equation $\Delta\;\mathrm{E}=\sqrt{{\left({\Delta \mathrm{L}}^{\ast}\right)}^2+{\left(\Delta\;{\mathrm{a}}^{\ast}\right)}^2+{\left({\Delta \mathrm{b}}^{\ast}\right)}^2}$, where ΔL*, Δa*, Δb* - difference between color attributes of sample with spirulina and control sample; *n* = 50

Table 4 shows the results of selected physical features of the prepared extrudates, grouped as expansion, texture and density indices. These features are mutually dependent and highly related to the sensory characteristics of puffed products [22]. Expansion is one of the most important properties of food products obtained through high temperature and low moisture extrusion cooking [23]. Proper expansion promotes dehydration and the development of a desirable crispy texture of the final extrudate. Therefore, expansion-related parameters are important to determine the quality of the extruded product [23]. For prepared extrudates, the radial expansion varied from 2.02 (control sample) to 1.62 cm (extrudates with 8% of spirulina and 2% of baking powder), while longitudinal expansion from 3.02 (control sample) to 2.40 cm (extrudates with 4% of spirulina). The calculated expansion ratio was the highest for the control sample (4.50) and decreased gradually with an addition of spirulina powder, reaching 3.59 for the variant with 8% of spirulina and 2% of baking powder. Expansion is a complex phenomenon which occurs as a consequence of several factors and mechanisms, influenced by feed composition and extrusion processing parameters [23]. Reduced expansion of extrudates enriched with spirulina may be the expected effect of an increased content of protein, since protein and starch compete for water and, in this case, starch gelatinisation is diminished or delayed [24].

**Table 4:** Expansion characteristics and hardness of extrudates

Physical feature	Extrudates											
	Control		2% S		4% S		6% S		8% S		8% S + 2% BP	
	x	SD	x	SD	x	SD	x	SD	x	SD	x	SD
Longitudinal expansion (cm)	3.02	0.20	2.65	0.17	2.40	0.09	2.41	0.10	2.53	0.13	2.91	0.15
Radial expansion (cm)	2.02	0.10	1.94	0.11	1.82	0.07	1.75	0.09	1.66	0.07	1.62	0.07
Expansion ratio (−)	4.50	0.30	4.31	0.23	4.05	0.16	3.89	0.19	3.70	0.17	3.59	0.17
Specific density (g/cm^3^)	0.082	0.001	0.083	0.02	0.085	0.02	0.098	0.01	0.097	0.01	0.083	0.02
Hardness (N)	37.29	13.27	36.25	9.66	35.20	7.97	34.42	8.54	33.74	9.17	34.58	11.08
Breaking stress (kPa)	6.11	0.49	7.05	0.34	8.06	0.51	8.16	0.43	8.03	0.41	7.34	0.43

*S* spirulina powder, *BP* baking powder, *WAI* water absorption index; *n* = 50

Density is the next important physical property of extrudates and is generally highly inversely related to expansion values. The determined density was in the range of 0.082–0.098 g/cm^3^ (Table 4). These results indicate the very light, puffed structure of all obtained extrudates. The highest bulk density values were found in the 6% and 8% spirulina supplemented samples (0.097–0.098 g/cm^3^), which were characterized by the lowest expansion. The opposite effect of spirulina addition (for enriched rice-soy crisp) was found by Bashir et al. [10], who determined the decrease in bulk density, from 0.356 (0% of spirulina) to 0.260 g/cm^3^ (8% of spirulina). In a study conducted by Joshi et al. [9], the bulk density of corn extrudate with 7.5% of spirulina was between 0.18 and 0.23 g/cm^3^ for a longitudinal expansion from 163 to 130%. This indicates that the observed effect of spirulina addition is highly variable and may be related to the used processing parameters (*e.g*., extruder type, moisture, temperature, pressure, etc.). However, the high differences in density determined in various studies are surprising. These values may vary from approximately 0.07 g/cm^3^ [25] to 0.79 g/cm^3^ [24] for similar expansion ratios.

The texture of extrudates was determined by measuring: 1) the force (N) required to destroy the extrudate structure and 2) the breaking stress, calculated as the value of peak force per cross-section area of extrudate. It is well-known that the increase in force/breaking stress indicates an increase in extrudate hardness [16]. The hardness of experimental extrudates only slightly varied between variants, reaching the mean values between 37.3 and 33.7 N (Table 4). The control sample was the hardest with a progressive increase of spirulina additive, which caused a tendency to increase softness. A similar phenomenon was noted by Morsy et al. [16] who found that the replacement of corn flour with up to 10% of spirulina improved the texture parameters in all produced extrudates. In contrast, a study conducted by Joshi et al. [9] showed that corn extrudates made with an addition of 7.5% of spirulina were harder than those made of pure corn. Despite these inconsistencies, the hardness and breaking stress are perceptible to consumers and were generally negatively correlated with the expansion and cell structure of the product, independent of the feed moisture content [16, 17, 26].

The water sorption properties of food are important for selecting suitable packaging materials and predicting the stability and moisture changes during storage. An increase in moisture content can directly affect the extrudate crispness, which is a key factor in their acceptance by consumers. As expected, the sorption of moisture by the tested extrudates increased during storage with increasing water activity (a_w_) from 0.07 to 0.98 (Table 5). Enriched extrudates stored at a_w_ lower than 0.54 were less moisturized, while those stored at a_w_ = 0.69 and higher were of higher moisture content. It points that spirulina components favor water absorption at higher relative humidity. The calculated values of energy constant *c*_*e*_ ≥ confirmed the sigmoidal shape of the sorption curves and suggested that only a process of physical sorption occurred in the tested products [27]. Based on the obtained values of the monolayer capacity (*v*_*m*_), the sorption surface and capacity of the capillaries were calculated and it was found that the highest sorption specific surface was for the variant with 2% of spirulina, while the highest total capillary capacity was for the variant with 8% of spirulina and 2% of baking powder. The size of the most likely radius capillary of the tested extrudates ranged from 2.88 to 3.52 nm. According to the study of Ocieczek [18], the size of the sorption surface is the resultant of the total surface and its affinity for water molecules, which is determined by the distribution of hydrophilic functional groups. The use of spirulina only slightly varied the water sorption characteristics. This suggests that the obtained products will behave in a similar manner during storage.

**Table 5:** Sorption characteristics of extrudates

Parameter	Extrudates					
	Control	2% S	4% S	6% S	8% S	8% S + 2% BP
Sorption specific surface (m^2^/g)	204.84	211.13	205.11	189.92	195.51	195.02
Total capillary capacity (nm^3^/100 g d.m.)	70.76	77.04	79.15	77.79	78.50	83.60
Capillary size a_w_ = 0.75 (nm)	2.88	3.49	2.97	2.96	2.96	3.52
Water content (g/100 g d.m.) after 45 days of storage at a_w_ = 0.07–0.98						
0.07	6.75	6.60	4.89	4.85	4.87	4.70
0.11	7.71	7.60	6.40	5.74	5.67	5.50
0.23	8.11	8.82	7.57	7.35	7.25	7.94
0.33	8.82	8.87	8.37	7.71	7.95	7.69
0.44	9.06	9.92	9.23	9.16	8.91	8.66
0.54	10.13	12.12	10.31	10.17	10.12	9.85
0.69	12.46	13.44	12.80	12.77	12.94	12.90
0.75	13.66	16.51	14.05	14.00	14.02	16.68
0.85	16.72	19.59	17.40	17.28	17.48	18.22
0.93	19.90	19.77	20.02	20.24	19.86	20.02
0.98	20.47	21.16	27.69	26.28	27.14	28.68
Parameters of BET equation						
*v*_*m*_	5.83	6.01	8.84	5.41	5.57	5.55
*c*_*e*_	79.88	90.06	87.57	125.91	76.89	83.70
R^2^	0.99	0.99	0.93	0.93	0.98	0.84
FitStdErr	0.74	0.70	0.48	0.43	0.27	0.78
F stat	2.09	5.31	27.43	27.67	81.64	10.74

*v*_*m*_ monomolecular layer capacity, *c*_*e*_ energy constant, *R* ^*2*^ determination coefficient, *FitStdErr* standard error, *Fstat* statistic F, *S* spirulina powder, *BP* baking powder; *n* = 3

## Conclusions

The results of the study indicated the successful incorporation of up to 8% of spirulina powder into corn extrudates. The highest amounts of spirulina only slightly decreased consumer acceptance of extrudates. This was compensated by the beneficial dietary effects, observed as an increase of protein, fat, fiber, and ash contents. However, phycocyanin was destroyed during extrusion, while retention of carotenoids and chlorophyll was up to 52 and 37%, respectively. The higher content of carotenoids and chlorophylls resulted in a visible change in the color of the extrudates, causing a decrease in their lightness and an increase of greenness and yellowness. Despite the gradual expansion decrease with an increase of spirulina addition, a tendency toward softness was observed in extrudates with higher spirulina content. The addition of spirulina only slightly varied the water sorption properties, which indicates the comparable storage stability as for the extrudates made from pure corn grits. The addition of baking powder had a strong protective effect on chlorophyll a and β-carotene during the extrusion process and reduced the extrudate hardness.
